# RNA-binding Protein MBNL2 regulates Cancer Cell Metastasis through MiR-182-MBNL2-AKT Pathway

**DOI:** 10.7150/jca.62816

**Published:** 2021-09-21

**Authors:** Guanglan Lin, Jiao Li, Jin Cai, Haowei Zhang, Qilei Xin, Ningchao Wang, Weidong Xie, Yaou Zhang, Naihan Xu

**Affiliations:** 1 State Key Laboratory of Chemical Oncogenomics, Tsinghua Shenzhen International Graduate School, Tsinghua University, Shenzhen 518055, China.; 2 Institute of Biopharmaceutical and Health Engineering, Tsinghua Shenzhen International Graduate School, Tsinghua University, Shenzhen 518055, China.; 3 Department of Neurology, Wuhan Hankou Hospital, Wuhan 430010, China.

**Keywords:** MBNL2, RNA-binding protein, miR-182, PI3K/AKT, Cancer, Metastasis

## Abstract

The aberrant expression of RNA-binding proteins (RBPs) plays important roles in the occurrence and progression of cancer. MBNL2 is a member of the RNA binding protein MBNL family that is widely expressed in mammalian cells. We report here that MBNL2 is downregulated in breast, lung and liver cancer tissues, the promoter methylation levels of MBNL2 are higher in cancer tissues than normal tissues. The enrichment analysis of MBNL2 correlated genes indicates the potential function of MBNL2 on cancer progression. MBNL2 regulates cancer cell migration and invasion by modulating PI3K/AKT-mediated epithelial-mesenchymal transition. PI3K/AKT inhibitor overcomes the promotive effect of shMBNL2 on metastasis. The expression of MBNL2 is directly targeted by miR-182. miR-182 is upregulated in breast, lung and liver cancers and has good potential for cancer diagnosis. miR-182 promotes cancer cell migration and invasion by inhibiting the expression of MBNL2. Re-introduction of exogenous MBNL2 reverses the promotive effect of miR-182 on metastasis. Collectively, these findings suggest that MBNL2 plays a tumor suppressive function through miR-182-MBNL2-AKT-EMT signaling pathways.

## Introduction

RNA-binding proteins (RBPs) plays central roles in post-transcriptional regulation. RBPs can interact with various RNAs (mRNAs, ncRNAs, tRNAs, *etc.*) to control all steps of RNA metabolism, such as microRNA processing, alternative splicing and polyadenylation, subcellular localization, stability, and translation of RNAs 1-3. Dysregulated RBPs are associated with various human diseases, including cancer. Several RBPs are predominantly up- or down-regulated in human cancers and show distinct molecular characteristics and prognostic effects 4,5. Aberrant expression of RBPs have been implicated in controlling the expression of RNAs related to cancer cell proliferation, apoptosis, angiogenesis, senescence, and epithelial-mesenchymal transition (EMT), ultimately contributing to oncogenesis and cancer development 6-9. Recent studies demonstrate that RBP gene-related signature has good predictive value and may have good prospects for improving clinical treatment and patient prognosis 10-12. Given their crucial roles in cancer, RBPs have emerged as promising targets of cancer therapeutics.

Muscleblind-like (MBNL) proteins are conserved multifunctional RNA-binding proteins which regulate tissue specific alternative splicing, mRNA stability and trafficking, alternative polyadenylation, as well as microRNA biogenesis 13-17. The MBNL family consists of three closely related paralogs in mammals, MBNL1, MBNL2, and MBNL3. MBNL1 and MBNL2 are ubiquitously expressed, whereas MBNL3 is predominantly in the placenta 18,19. MBNL family plays important roles in the differentiation of embryonic stem cells, as well as in neuronal differentiation. MBNL deficient mice develop myotonia and skeletal myopathy, key features of human myotonic dystrophy (DM) 20-23. Loss of function of MBNL1/2 also causes fundamental defects in cortical neuron distribution and morphological changes in dendritic spines and postsynaptic densities 24.

Recent studies indicate that MBNL family proteins also participate in the occurrence and progression of cancer. MBNL1-mediated alternative mRNA splicing patterns contribute to the pathogenesis of MLL-rearranged leukemias 25. MBNL1 inhibits glioma stem cell self-renewal and tumorigenic potential, hypoxic response within the tumor inhibits MBNL1 activity, promoting stem-like phenotypes and tumor growth 26. MBNL1 is downregulated in several common cancers, low MBNL1 is correlated with poor overall survival and increased relapse and distant metastasis 27. MBNL1 also inhibits tumor cell metastasis through regulating the stability of Snail, TACC1 and DBNL 28,29. The role of MBNL2 on cancer is contradictory. MBNL2 is upregulated in metastatic renal carcinoma tissues and drives the abnormal cancer-associated transcripts, suggesting an oncogenic function of MBNL2 30. Chronic hypoxia specifically induces MBNL2, MBNL2 drives cancer cell proliferation and migration by regulating the expression and alternative splicing of hypoxia-induced genes, such as VEGFA 31. In contrast, some studies indicate that MBNL2 possesses antitumor activity. MBNL2 expression level is negatively correlated with tumor size and stage in hepatocellular carcinoma, overexpression of MBNL2 inhibits liver cancer cell growth and invasion both *in vitro* and in vivo 32. MBNL2 increases the anticancer ability of a natural compound Neobractatin 33. MBNL2 also regulates tumor cell proliferation and DNA damage response by stabilizing p21 34.

In this report, we further explore the molecular mechanism underlying the tumor suppressive function of MBNL2 in a panel of human cancers. We show that MBNL2 is downregulated in breast, liver and lung cancers, overexpression of MBNL2 inhibits cancer cell metastasis by modulating PI3K/AKT mediated EMT process. High levels of MBNL2 promoter methylation and miR-182 may contribute to decreased MBNL2 expression in cancer tissues. miR-182 directly targets the expression of MBNL2, exogenous MBNL2 can overcome the promotive effect of miR-182 on cancer cell metastasis. Thus, we propose that MBNL2 plays tumor suppressive function by modulating miR-182-MBNL2-AKT-EMT signaling pathway.

## Materials and Methods

### Cell culture and transfection

MDA-MB-231, A549, MCF7, HEK 293T and HepG2 cells were obtained from American Type Culture Collection (Manassas, VA, USA). Cells were grown in Dulbecco's modified Eagle medium (Thermo Fisher Scientific, Waltham, MA, USA), supplemented with 10% fetal bovine serum (Biowest, France) and 1% Antibiotic-Antimycotic (Thermo Fisher Scientific, Waltham, MA, USA). To generate stable MBNL2 knockout cells, shMBNL2 lentiviral constructs were purchased from Genechem and infected MDA-MB-231, A549 and HepG2 cells. To generate stable MBNL2-overexpressed cell lines, pVSVG (Addgene #8454), psPAX2 (Addgene #12260), PCDH-EF1and MBNL2-PCDH-EF1 (Unibio) plasmids were constructed, and HEK 293T cell used to generate high titer lentivirus. The lentivirus-infected cell lines and plasmid were cultured under the selection pressure of 2 μg/mL puromycin (Thermo Fisher Scientific, Waltham, MA, USA). Lipofectamine 2000 (Invitrogen Life Technologies, Waltham, MA, USA) was used to transfect miRNA, miRNA inhibitor or siRNA.

### Bioinformatics Analysis

The expression signatures of MBNL2 were investigated in BRCA, LIHC, LUAD, LUSC samples from the TCGA database (https://www.cancer.gov) and GEO database (lung cancer chip GSE19804 and breast cancer chip GSE10797) (https://www.ncbi.nlm.nih.gov/geo/). The promoter methylation level of MBNL2 in BRCA, LIHC, LUAD, LUSC were investigated by UALCAN database (http://ualcan.path.uab.edu/). The correlation between survival and MBNL2 expression was analyzed by Kaplan-Meier Plotter database (https://kmplot.com/analysis/).

For enrichment analysis of MBNL2 related signaling pathways, MBNL2 was used as the target gene to search for similar genes in GEPIA, according to the pearman correlation coefficient (PCC), the top 1000 genes were selected in BRCA, LIHC and LUAD and download the gene list, the three gene lists were put into Metascape for pathway enrichment analysis, Metascape depicts top enriched clusters and their enrichment patterns as a clustered heatmap.

To investigate potential microRNAs target MBNL2, we identified 78 potential miRNAs by combining four well-recognized miRNA databases, mirDIP (http://ophid.utoronto.ca/mirDIP/), TargetScan (http://www.targetscan.org/vert_72/), miRDB (http://mirdb.org) and miRWalk (http://zmf.umm.uni- heidelberg.de/apps/zmf/mirwalk2/). The BRCA-miRNA data were downloaded from TCGA and divided into high and low expression groups by R package limma, we identified 70 highly expression miRNAs and 82 lowly expression miRNAs. Then, 78 microRNAs identified from four microRNA databases were compared with the high and low expression miRNAs in TCGA BRCA data, the results showed that there were 9 up-regulated miRNAs and 5 down regulated miRNAs. Finally, the expression signatures of these miRNAs were investigated in BRCA, LIHC, LUAD, LUSC samples from the TCGA miRNA database.

### Transcriptome analysis

Transcriptome analysis was performed by Gene Denovo Biotechnology Co. (Guangzhou, China). Briefly, total RNAs in control or *MBNL2* knockout cells were extracted using Trizol reagent. Eukaryotic mRNA was enriched by Oligo (dT) beads and reversed transcribed into cDNA with random primers. The cDNA fragments were purified, end-repaired, poly A added, and ligated to Illumina sequencing adapters. The ligation products were sequenced using Illumina HiSeq^TM^ 2500. Differently expressed genes (DEGs) were identified with a fold change ≥ 2 and a false discovery rate (FDR) < 0.05 in a comparison as significant DEGs, DEGs were then subjected to enrichment analysis of GO functions and KEGG pathways. The Q value is an adjusted p value after multi-hypothesis test correction, taking FDR ≤ 0.05 as a threshold. The Richfactor refers to the ratio of the number of the differentially expressed to the total number of annotated genes, the larger the Richfactor value, the greater the degree of enrichment.

### Immunoblotting

Samples were collected and lysed in protein lysis buffer (5 mL 1 M Tris-HCl (pH 8.0), 26 g urea, 1 mL Triton X-100, 2 pieces of protease inhibitor cocktail, extended to the total volume of 100 mL with ddH2O). Protein samples were separated by SDS-PAGE and then transferred to nitrocellulose membrane (PALL, NY, USA). The membranes were incubated in primary antibodies (anti-MBNL2, ab171551, 1:750; anti-MBNL2, CST 29733S, 1:1000; anti-GAPDH, Proteintech 10442-1-AP, 1:10000; anti-ZEB1, ab203829, 1:500; anti-Vimentin, abD21H3, 1:500; anti-E-cadherin, CST 24E10, 1:1000; 1:500; anti-AKT, CST C67E7, 1:1000; anti-p-AKT(ser473), CST D9E, 1:1000; anti- PI3Kinase P110β, CST 3011, 1:1000; anti- PTEN, CST 138G6, 1:1000; anti- P-GSK3β, CST D85E12, 1:1000), followed incubation with secondary antibodies (1:5000, 52200336, SeraCare, Milford, MA, USA), Membrane was finally supplemented with the Pierce ECL Western Blotting Substrate (Thermo Fisher Scientific) and imaged with an autoradiography film (FUJIFILM, Minato, Japan).

### Migration and invasion assays

Cells were suspended in serum-free medium and seeded into the upper chamber of transwell insert membranes of an 8 μm pore size (Corning) or coated with Matrigel (Corning) in a 24-well plate (3-10×10^4^ cells per well). Approximately 10% fetal bovine serum (FBS) was used in the bottom chamber as the chemoattractant. The cells in the upper chamber were removed 24-72 h later using a cotton swab and washed one to two times with PBS and fixed by methanol and then stained with 0.5% crystal violet. The number of successfully translocated cells was counted under microscope.

### RNA isolation and quantitative RT-PCR

Total RNA was extracted using the RNAiso Plus Reagent (TaKaRa, Mountain View, CA, USA), from culture cells according to the manufacturer's instruction. The cDNA was synthesized from total RNA using ReverTra Ace qPCR RT Master Mix (TOYOBO, Osaka, Japan) or TaqMan™ MicroRNA Reverse Transcription Kit (Life Technologie). qPCR was performed with SYBR Green Realtime PCR Master Mix (TOYOBO, Osaka, Japan) and primer pairs on a qTOWER 2.2 (Jena, Germany). The level of mature miR-182 was normalized relative to U6 endogenous control and MBNL2 expression was normalized relative to GAPDH (endogenous control) by the 2^-∆∆CT^ method. The primer of miR-182 and U6 purchased from Thermo Fisher. Primer sequences are listed as follows: MBNL2, forward primer 5′-TCAAAGAGGAACATGCTCACG-3′, reverse primer 5′-AACGGCCCTTTAGGGAATCAA-3′; GAPDH, forward primer 5′-AAGGCTGTGGGCAAGG-3′, reverse primer 5′-TGGAGG AGTGGGTGTCG-3′.

### Luciferase reporter assay

The pGL3 luciferase reporters containing wild-type MBNL2 (WT) or mutant-type (MUT) miR-182 binding sites were constructed by YouBio (Changsha, China). For luciferase assay, miR-182 mimic plus WT or MUT 3'UTR of MBNL2 were transfected into MDA-MB-231 cells by lipofectamine. After 48-72 hours, the cells were collected and firefly and renilla luciferase activities were measured with Promega Dual-Luciferase® Reporter Assay System (Promega Corporation, Madison, WI, USA).

### Immunohistochemistry

The breast cancer, liver cancer and lung adenocarcinoma tissue microarray purchased from SHANGHAI OUTDO BIOTECH CO.LTD, the array numbers are HBre-Duc060CS-04, HLivH060CS01 and HLugA060PG02 respectively. The primary antibodies for IHC staining are mouse monoclonal Ab against human MBNL2 (sc-136167, Santa Cruz Biotechnology, CA, USA). The paraffin-embedded slide was deparaffinized, rehydrated, and block in sheep serum for 30 min, followed by incubation with anti-MBNL2 overnight at 4 °C. The slide was mounted with D.P.X. for histology analysis. A semi-quantitative method based on the staining intensity and the area of positive cells was used. The staining scores were calculated by multiplying the above two parameters. The MBNL2 staining results were assessed according to the staining scores.

### Statistical analysis

Student's t test, the Mann-Whitney U test were used to analyze differences among different groups. All the results were presented as the mean ± SD and P values < 0.05 were considered statistically significant.

## Results

### MBNL2 is downregulated in some human cancers

**Figure 1:**
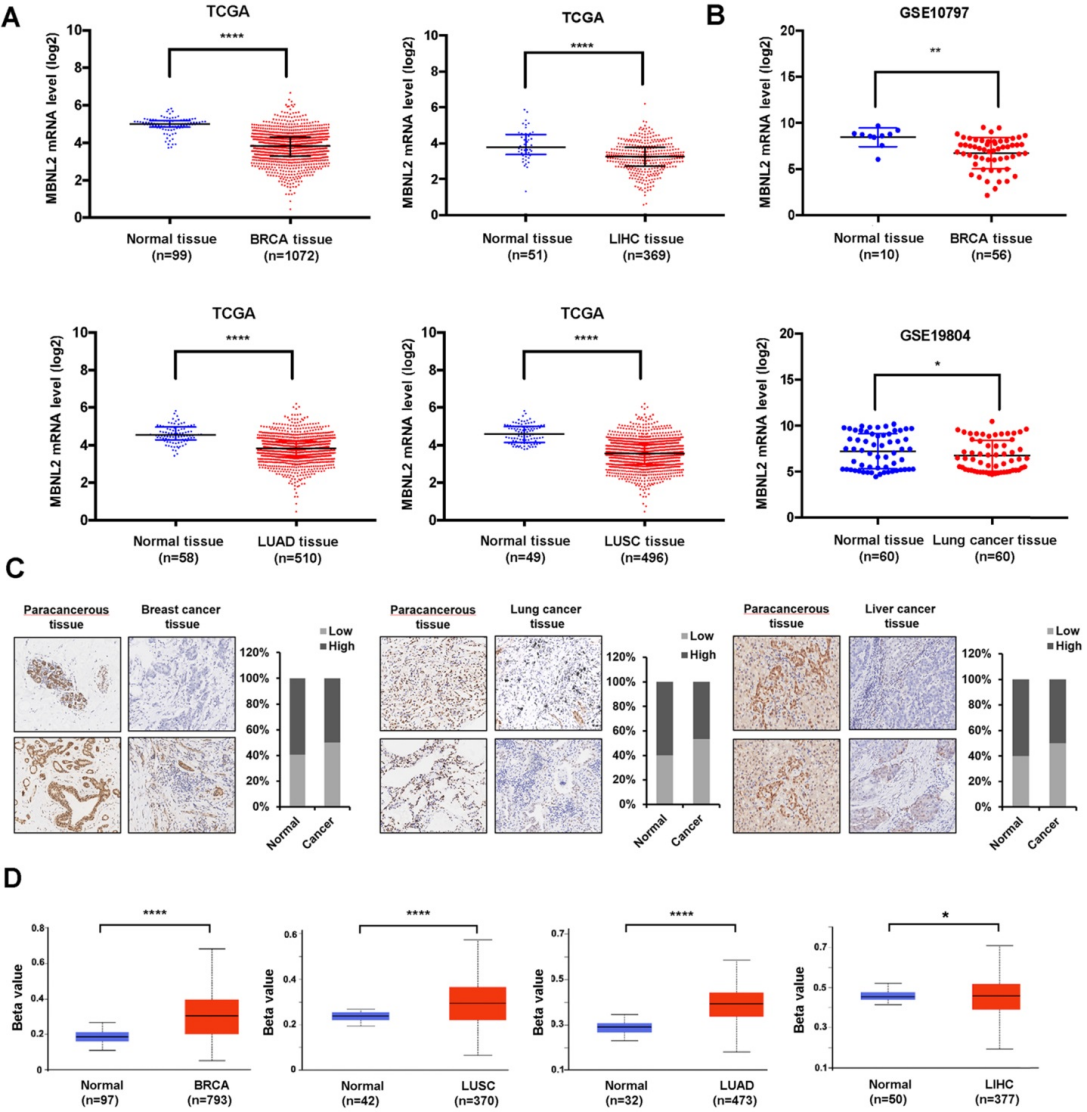
** Analysis the expression of MBNL2 in breast, lung and liver cancers. (A)** Scatter diagram derived from gene expression data in TCGA comparing the expression of MBNL2 in tumor tissue and normal tissue in BRCA, LIHC, LUAD and LUSC, ****p<0.0001. **(B)** Scatter diagram derived from gene expression data in GEO datasets (GSE10797 and GSE19804) comparing the expression of MBNL2 in tumor tissue and normal tissue in breast and lung cancers, *p<0.05, **p<0.01. **(C)** Representative images of IHC staining in human breast, lung and liver cancer tissue with MBNL2 antibody. **(D)** Promoter DNA methylation level of MBNL2 in BRCA, LUAD, LUSC and LIHC, data from UALCAN, *p<0.05, ** p<0.01, ****p<0.0001.

To study the function of MBNL2 in human cancers, we analyzed TCGA RNA-seq data sets. A significant downregulation of MBNL2 was observed in several cancer types, including breast cancer, liver cancer, lung adenocarcinoma, and lung squamous cell carcinoma (Figure 1A). The microarray data from GEO also showed downregulation of MBNL2 in breast and lung cancer tissues (Figure 1B). We detected MBNL2 protein expression by immunohistochemistry in commercial tissue microarrays which containing cancer tissues of patients with primary operable breast cancer, lung cancer, or liver cancer and adjacent normal tissues (Table 1). MBNL2 protein was absent or weakly detected in cancer tissues, whereas adjacent normal tissues expressed higher levels of MBNL2 (Figure 1C).

Aberrant promoter methylation is considered a hallmark of cancer involved in silencing of tumor suppressor genes and activation of oncogenes. We analyzed MBNL2 promoter methylation levels in different cancer types. Methylation indices for MBNL2 in BRCA, LIHC, LUSC and LUAD patients were found to be significantly higher as compared to that in normal individuals (Figure 1D). These findings indicate increased methylation of MBNL2 promoter may contribute to reduced gene expression in cancer.

### MBNL2 regulates cancer cell migration and invasion

**Figure 2:**
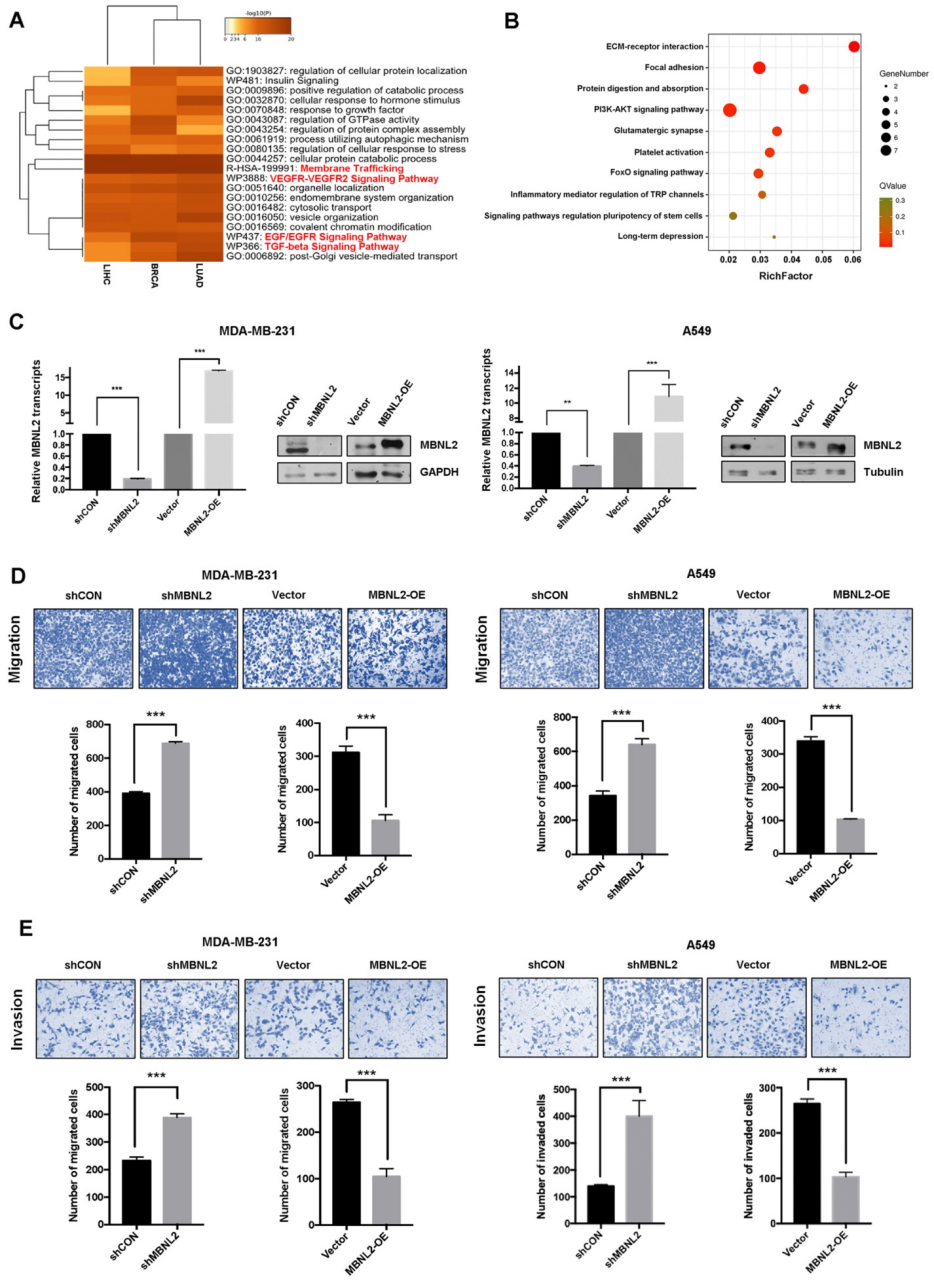
** MBNL2 regulates cancer cell migration and invasion. (A)** Metascape depicts top enriched clusters and their enrichment patterns across multiple gene lists (MBNL2 correlated genes in BRCA, LUAD and LIHC, data from GEPIA) as a clustered heatmap.** (B)** KEGG pathway enrichment analysis of RNA-seq data from cells transfected with empty vector or His-MBNL2. **(C)** MBNL2 mRNA and protein expression in MDA-MB-231 and A549 cell lines measured by qRT-PCR and Western blotting. **(D)** Migration assays and **(E)** Invasion assays showing that overexpression of MBNL2 inhibited the ability of migration and invasion in MDA-MB-231 and A549 cells, knockdown of MBNL2 increased the number of migrated and invaded cells. ***p<0.001.

To explore the biological function of MBNL2 in cancer, we identified the top 1000 MBNL2 correlated genes from TCGA breast, lung and liver cancer data sets and the gene lists were submitted to Metascape to perform gene ontology enrichment analysis. According to GO term analysis, genes correlated with MBNL2 were significantly enriched for biological processes associated with membrane trafficking, TGF-beta signaling, VEGFR and EGF/EGFR signaling, which are all related to cancer cell invasion and metastasis (Figure 2A, Supp Figure 1). We overexpressed MBNL2 in cancer cells and performed transcriptome and gene expression analysis. GO and KEGG pathway enrichment analysis revealed that ECM-receptor interaction, focal adhesion and PI3K-AKT signaling are among the top five enriched pathways, these pathways are closely associated with cancer metastasis (Figure 2B). Therefore, we hypothesized that MBNL2 might function as a tumor suppressor in breast, liver and lung cancers. To this end, we knocked down MBNL2 expression by using shRNA, or overexpressed His-MBNL2 in MDA-MB-231 breast cancer cells, A549 non-small-cell lung cancer cells, and HepG2 liver cancer cells, the knock-down or knock-in efficiency were verified by qRT-PCR and western blot (Figure 2C). Transwell migration assay and matrigel invasion assay revealed that overexpression of MBNL2 dramatically reduced the number of migrated and invaded cells, whereas MBNL2 shRNA showed the opposite effects (Figure 2D and E, supp Figure 2A and B).

### MBNL2 regulates EMT process through PI3K/AKT signaling

**Figure 3:**
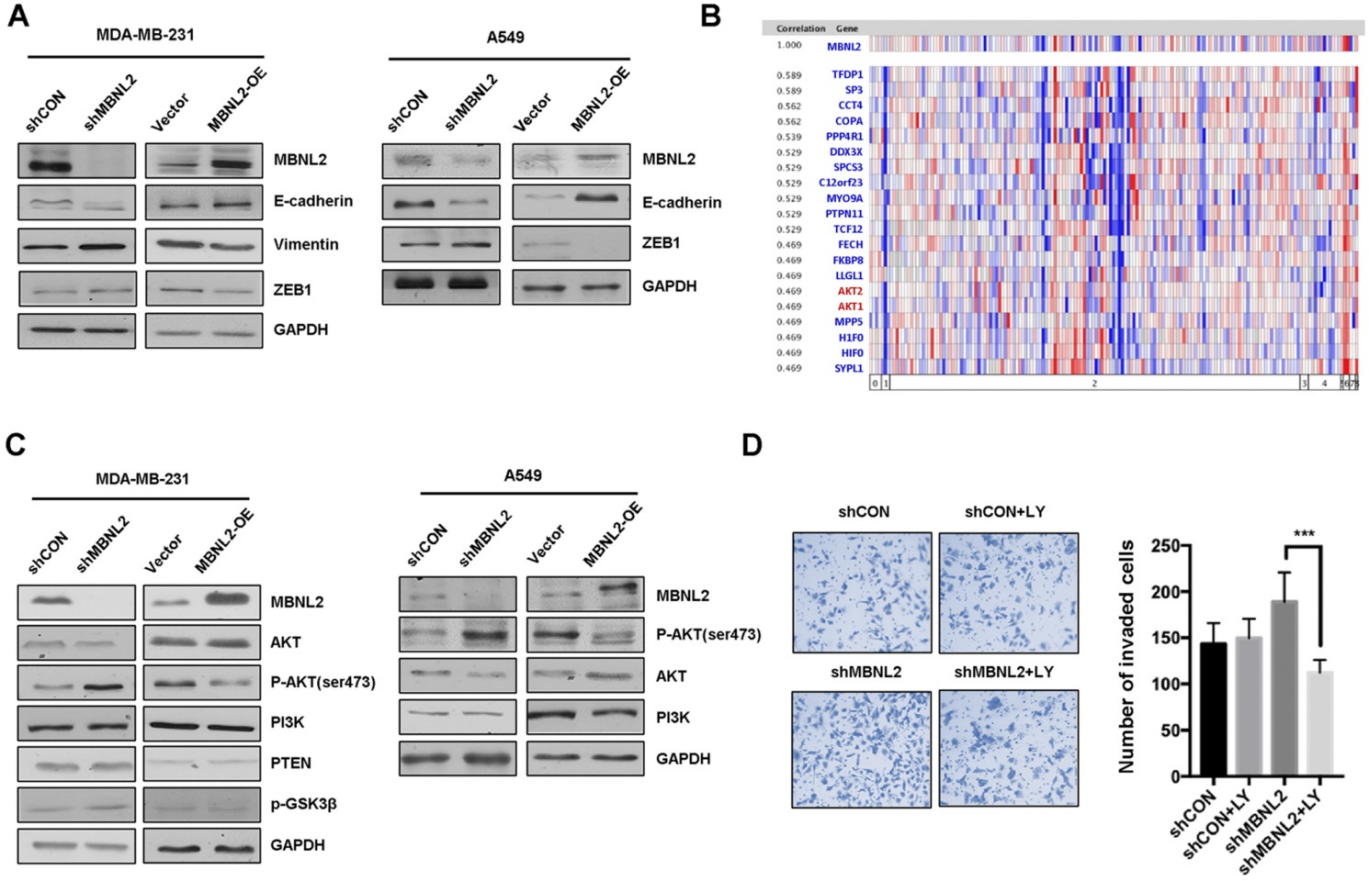
** MBNL2 regulates EMT through PI3K/AKT signaling. (A)** Western blot analysis of EMT related markers (E-cadherin, Vimentin and ZEB1) in MDA-MB-231 and A549 cells overexpressing or silencing MBNL2. **(B)** Oncomine analysis showing the expression of MBNL2 was significantly associated with AKT1 and AKT2. **(C)** Western blot analysis of PI3K/AKT related marker (AKT, p-AKT, PI3K, PTEN and p-GSK3β) in MDA-MB-231 and A549 cells overexpressing or silencing MBNL2. **(D)** AKT inhibitor LY294002 inhibits shMBNL2-induced cell invasion in MDA-MB-231 cells.

Epithelial-mesenchymal transition (EMT), an evolutionarily conserved developmental process, is well recognized as a key step in cancer invasion and metastasis. Therefore, we investigated whether MBNL2 regulates metastasis through EMT in MDA-MB-231, A549 and HepG2 cells. Western blotting revealed that knockdown of MBNL2 downregulated the expression of epithelial marker E-cadherin and upregulated the expression of mesenchymal markers Vimentin and ZEB1, however, overexpression of MBNL2 showed the opposite effects (Figure 3A, supp Figure 2C).

To further study the molecular mechanism of MBNL2 on EMT, we performed gene coexpression analysis using the Oncomine breast cancer databases. The results showed that both AKT1 and AKT2 are among the top 20 genes that are highly coexpressed with MBNL2 (Figure 3B). Because the role of PI3K/AKT pathway in EMT is well recognized, thus we aimed to study whether MBNL2 regulates EMT through PI3K/AKT. Immunoblotting revealed that knockdown of MBNL2 increased the level of phosphorylated AKT S473 without changing the protein expression of PI3K, AKT and PTEN, whereas exogenous MBNL2 inhibited the phosphorylation of AKT S473 in both MDA-MB-231 and A549 cells (Figure 3C). Treatment with PI3K inhibitor LY294002 in MBNL2 knockdown cells reduced the number of invaded cells (Figure 3D). Collectively, these data demonstrated that silencing MBNL2 promoted EMT process by activating PI3K/AKT signaling.

### MiR-182 is upregulated in some human cancers

**Figure 4:**
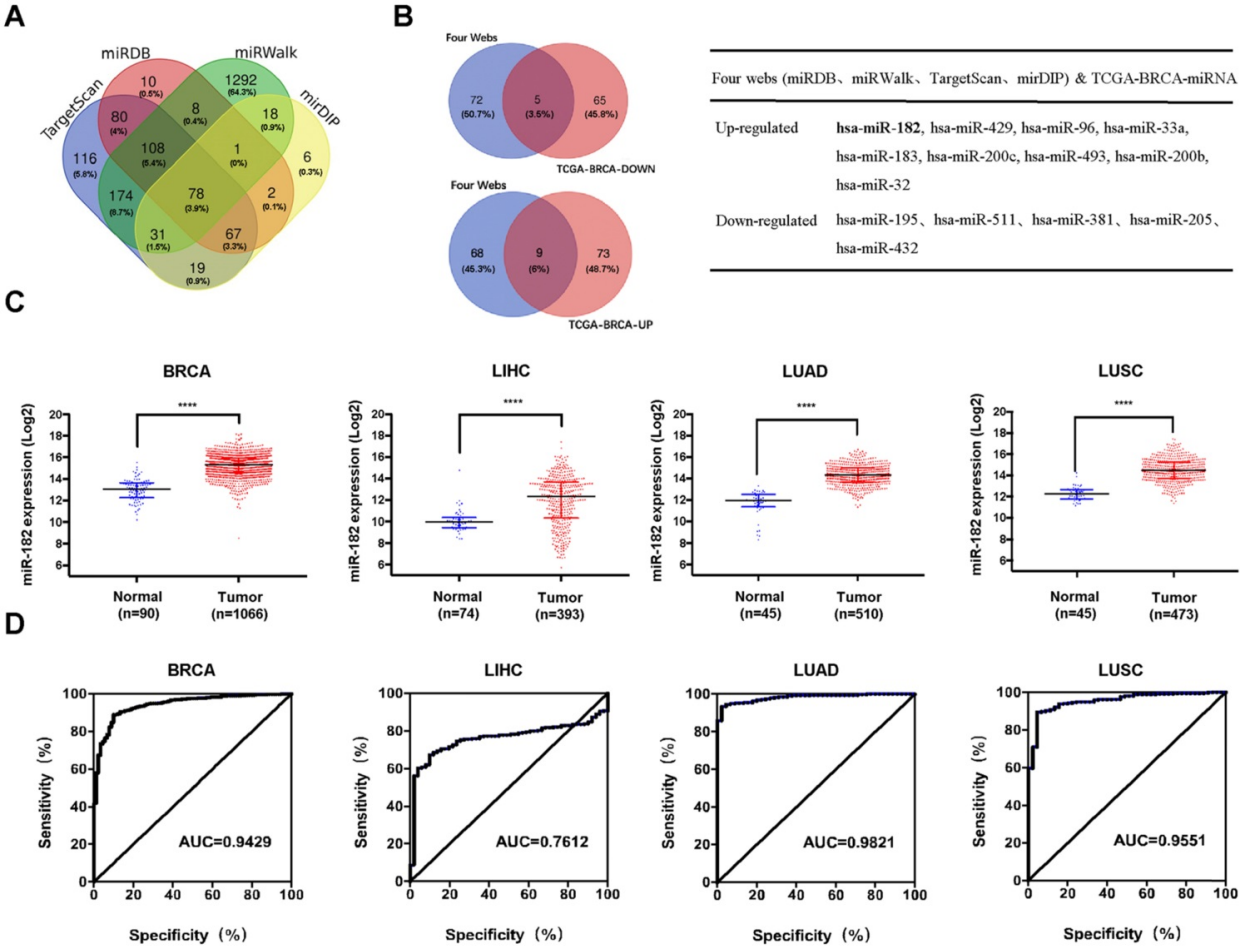
** Identification of miRNAs targeting MBNL2 in cancer. (A)** 78 miRNAs were predicted to target MBNL2 by four miRNA databases (Targetscan, miRDB, miRWalk and mirDIP). **(B)** Overlapping of 77 MBNL2-targeting miRNAs and TCGA-BRCA differentially expressed miRNAs, 9 MBNL2-targeting miRNAs were up-regulated and 5 MBNL2-targeting miRNAs were downregulated in breast cancer. **(C)** Scatter diagram derived from gene expression data in TCGA comparing the expression of miR-182 in tumor tissues and normal tissues in BRCA, LIHC, LUAD and LUSC, **** p<0.0001. **(D)** ROC curve analysis. The AUC was analyzed to evaluate the potential of miR-182 in cancer diagnosis. The x-axis indicated specificity (%) and y-axis indicated sensitivity (%).

We were keen to understand the upstream signals responsible for the decreased levels of MBNL2 in human cancers. MicroRNAs generally bind to the 3'UTR of their target mRNAs and suppress protein production through post-transcriptional mechanisms, we therefore explored the miRNAs targeting MBNL2 using four miRNA target prediction databases, miRDB, mirDIP, miRWalk, and TargetScan. A total of 2010 miRNAs were predicted, among which 77 miRNAs were common to all four algorithms (Figure 4A). Because MBNL2 is downregulated in breast cancer tissues, we further identified MBNL2-targetting miRNAs which were upregulated in breast cancer. Analysis of TCGA breast cancer miRNA-seq data revealed 82 upregulated and 70 downregulated miRNAs in breast cancer. The number of MBNL2-targeting miRNAs that were up or down regulated in breast cancer is represented in Venn diagrams. Finally, we obtained 9 MBNL2-targeting miRNAs that were upregulated in breast cancer, including miR-182, miR-429, miR-97, miR-33a, miR-183, miR-200c, miR-493, miR-200b and miR-32 (Figure 4B). We analyzed the expression levels of these upregulated miRNAs in other human cancer types, the results showed that only miR-182 were upregulated in breast, liver and lung cancers (Figure 4C). In receiver operating characteristic (ROC) curve analysis, the area under the curve (AUC) is the most widely used index of diagnostic accuracy for assessing the sensitivity and specificity of a biomarker. We therefore performed ROC curve analysis to evaluate the application of miR-182 in clinical diagnosis. The results indicated that miR-182 had a good performance in the diagnosis of breast, lung and liver cancers (Figure 4D).

### MiR-182 targets the expression of MBNL2

**Figure 5:**
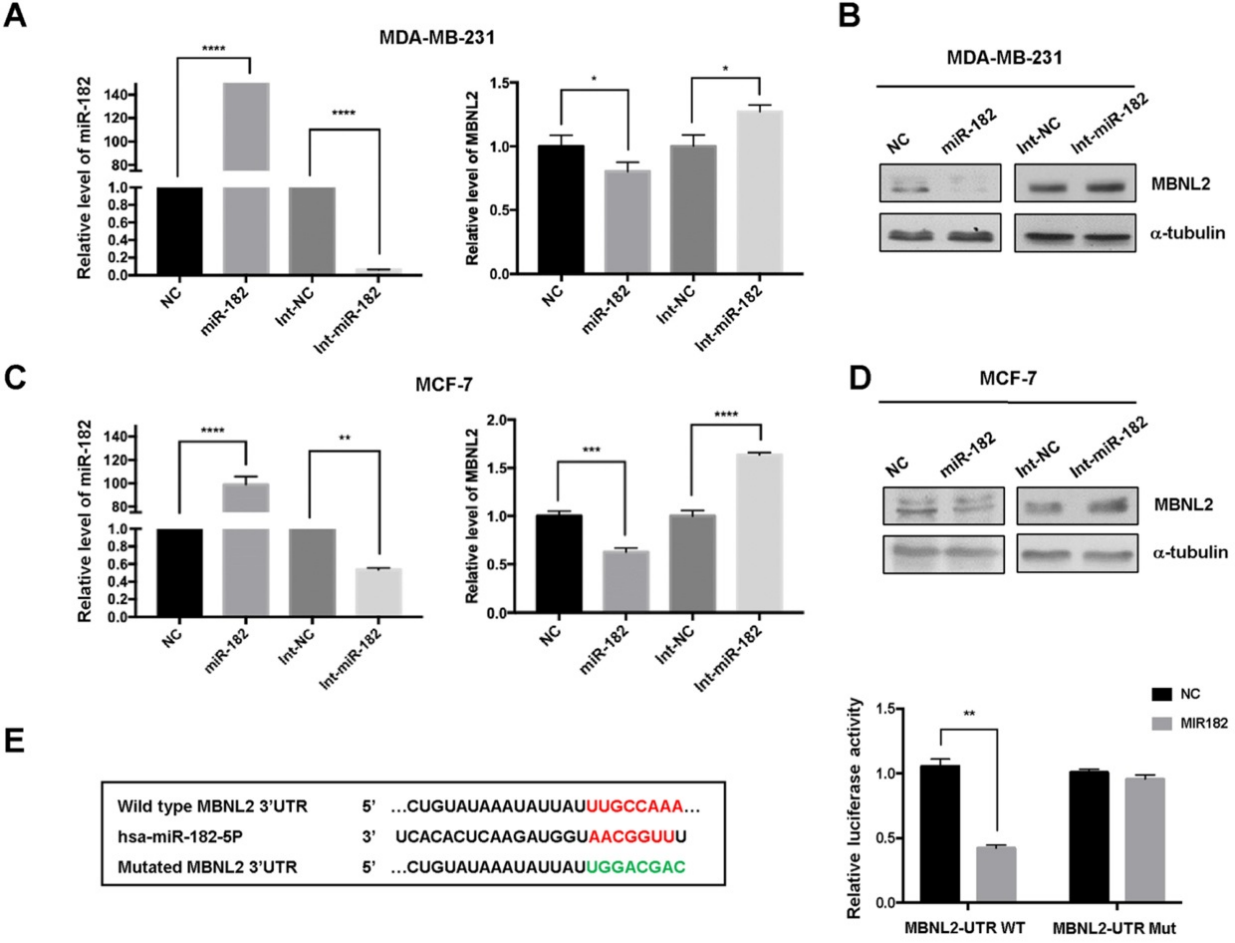
** miR-182 directly targets the expression of MBNL2. (A)** Relative mRNA levels of miR-182 and MBNL2 in MDA-MB-231 and **(C)** MCF-7 cells overexpressing NC, miR-182 mimics, NC inhibitor (Int-NC) and miR-182 inhibitor (Int-miR-182). **(B)** The protein levels of MBNL2 were analyzed in in MDA-MB-231 and **(D)** MCF-7 cells transfected with indicated miRNAs and inhibitors. **(E)** Schematic illustration of the predicted miR-182 binding site in MBNL2 3'UTR. Luciferase reporters containing the wile type (WT) or the mutated (Mut) MBNL2 3'UTR were co-transfected with either NC or miR-182 into MDA-MB-231 cells, dual luciferase assay was performed and normalized to NC. **p<0.01.

To validate the effect of miR-182 on MBNL2 expression, we performed quantitative RT-PCR analysis. Overexpression of miR-182 reduced the transcript abundance of MBNL2 in both MDA-MB-231 and MCF-7 cells, knockdown of endogenous miR-182 with inhibitor (Int-miR-182) increased the amounts of MBNL2 mRNA in both cells (Figure 5A, C). Immunoblotting results showed that miR-182 suppressed protein expression of MBNL2, whereas miR-182 inhibitor moderately increased MBNL2 expression in both MDA-MB-231 and MCF-7 cells (Figure 5B, D). Then, we performed luciferase assay to determine the regulatory effect of miR-182 on MBNL2 gene expression. miR-182 dramatically reduced the luciferase activity in MCF-7 cells transfected with luciferase reporter containing wild-type, but not mutated 3'UTR of MBNL2 (Figure 5E).

### MiR-182 controls tumor cell metastasis through MBNL2

**Figure 6:**
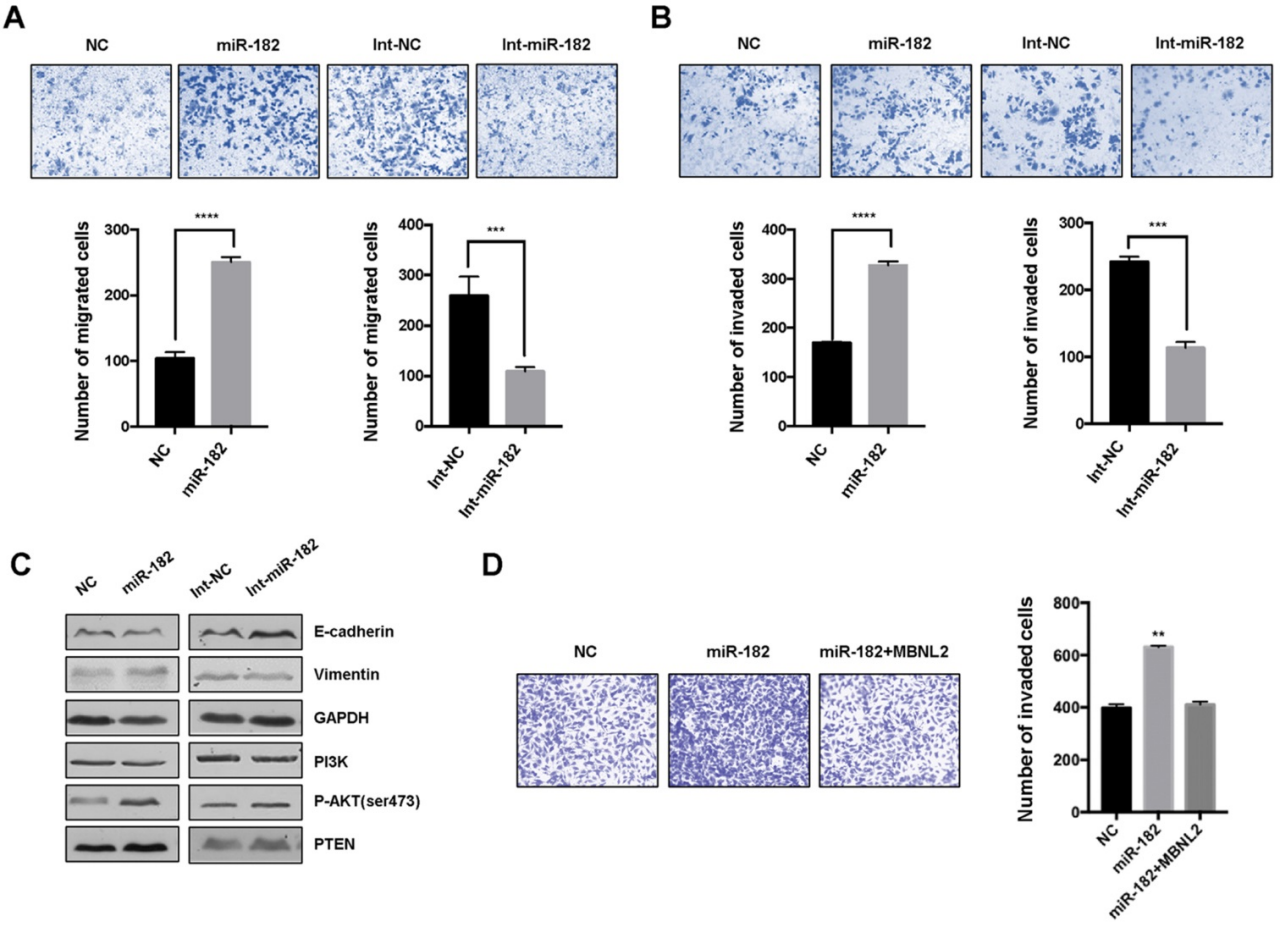
** miR-182 regulates cancer cell migration and invasion through MBNL2. (A)** Cell migration and **(B)** invasion assay showing the effect of miR-182 or miR-182 inhibitor on cancer metastasis. **(C)** Western blot analysis of EMT related markers (E-cadherin and Vimentin), PI3K, pAKT S473, and PTEN in MCF-7 cells transfected with either miR-182 mimics or inhibitor. **(D)** Invasion assays showing that overexpression of MBNL2 could rescue the promotive effect of miR-182 on cancer cell invasion.

We performed transwell migration assay and matrigel invasion assay to determine the effects of miR-182 on cancer cell metastasis. The results showed that overexpression of miR-182 mimics led to increased number of migrated and invaded cells, inhibition of endogenous miR-182 with miRNA inhibitor markedly attenuated migration and invasion in breast cancer cells (Figure 6A and B). Immunoblotting revealed that miRNA-182 mimics downregulated the expression of epithelial marker E-cadherin and upregulated the expression of mesenchymal markers Vimentin, whereas miR-182 inhibitor showed the opposite effects. Moreover, the level of phosphorylated AKT S473 was increased in cells overexpressing miR-182 mimics, indicating that miR-182 may regulate EMT process through AKT signaling. We also observed whether re-introduction of exogenous MBNL2 could provide resistance to miR-182 mediated cell metastasis. Invasion assay demonstrated that overexpression of MBNL2 was sufficient to rescue the promotive effect of miR-182 on cancer cell metastasis (Figure 6D).

## Discussion

Except the well-studied function in DM, recent reports suggested that MBNL2 also participates in tumorigenesis and tumor progression. However, the role of MBNL2 on cancer is controversial. Previously, we report that MBNL2 post-transcriptionally regulates p21 mRNA stability independently of p53, depletion of MBNL2 promotes colon cell proliferation 33. In this study, we further explored the molecular mechanism underlying the tumor suppressive function of MBNL2 in a panel of human cancer cells. We observed that MBNL2 is down regulated in breast, lung, and liver cancer. MBNL2 modulates multiple pathways involving tumorigenesis and tumor progression. Depletion of MBNL2 promotes whereas exogenous MBNL2 inhibits migration and invasion in breast, lung and liver cancer cells. We identified that MBNL2 modulates the activation of AKT without changing the protein expression of PI3K, AKT and PTEN. Treatment of PI3K/AKT inhibitor could overcome the promotive effect of shMBNL2 on cancer cell invasion. Together, these results further confirm the tumor suppressive function of MBNL2 in some types of human cancer.

MiRNAs control gene expression through a post-transcriptional mechanism. Human miRNAs are frequently located at fragile sites and chromosomal regions affected in cancer 35-36. miR-182 is one of the most frequently studied cancer-related miRNAs that is dysregulated in various human cancers, miR-182 may function as an oncogenic or tumor suppressor miRNA in different cancer types. Some studies report that miR-182 promotes cancer cell proliferation and invasion in hepatocellular carcinoma, prostate cancer, breast cancer and glioma 37-40. However, some studies show that miR-182 may function as tumor suppressor, miR-182 is frequently downregulated in gastric cancer and metastatic NSCLC, overexpression of miR-182 inhibits cell growth, EMT and metastasis 41-42. miR-182 sensitizes human ovarian cancer cells to cisplatin through direct targeting CDK6 43. miR-182 also inhibits colon cancer tumorigenesis, angiogenesis and lymphangiogenesis by targeting VEGF-C 44. These conflicting results suggest the role of miR-182 is complex in cancer. To identify the upstream signals responsible for downregulation of MBNL2 in cancer, we explored the MBNL2-targeting miRNAs upregulated in breast cancer tissues using TCGA miRNA-seq data. We show miR-182 is upregulated in breast, liver and lung cancer tissues. ROC curve analysis indicates miR-182 may serve as a valuable biomarker for diagnosis. miR-182 plays oncogenic function in breast cancer, lung and liver cancers. Overexpression of miR-182 promotes invasion through MBNL2, exogenous MBNL2 can rescue the inhibitory effect of miR-182 on metastasis. Since miR-182 directly targets the expression of MBNL2, the upregulation of miR-182 may contribute to the downregulation of MBNL2 in these cancer types.

The aberrant expression of RBPs have been implicated in various types of human cancers. DNA methylation has been widely described as the main epigenetic mechanism that plays a significant role in gene expression, X-chromosome inactivation, genetic imprinting, differentiation and development 45-48. Hypermethylation occurring in the context of the CpG at a promoter region prevents the binding of transcription factors, thereby inhibiting gene transcription 49. We show here the methylation status of MBNL2 promoter is significantly higher in tumor tissues comparing to normal tissues. Hypermethylation of MBNL2 is associated with downregulation of MBNL2 in tumor tissues. When taken together, we hypothesized that both DNA methylation and miR-182 may contribute to MBNL2 downregulation in cancer. MBNL2 plays a tumor suppressive function through miR-182-MNL2-AKT-EMT signaling pathway.

## Supplementary Material

Supplementary figures.

## Figures and Tables

**Table 1:**
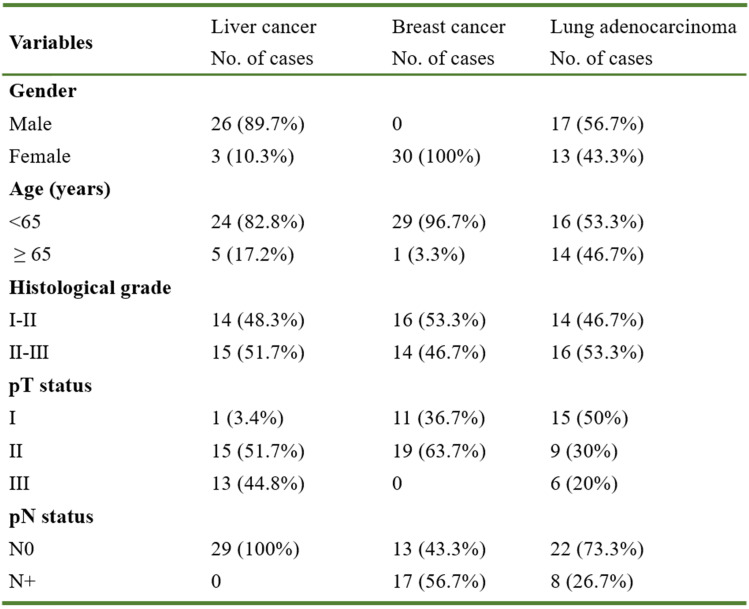
Basic clinicopathological factors of patients included

## Abbreviations

RBPs: RNA-binding proteins
MBNL2: muscleblind like splicing regulator 2
PI3K: phosphatidylinositol 3-kinase
KEGG: Kyoto Encyclopedia of Genes and Genomes
GEO: Gene Expression Omnibus
qPCR: quantitative PCR
siRNA: small interfering RNA
shRNA: short hairpin RNA
UTR: untranslated region
BRCA: breast cancer
LIHC: liver hepatocellular carcinoma
LUSC: lung squamous cell carcinoma
LUAD: lung adenocarcinoma

## References

[1] Lunde BM, Moore C, Varani G (2007). RNA-binding proteins: modular design for efficient function. Nat Rev Mol Cell Biol.

[2] Keene JD (2007). RNA regulons: coordination of post-transcriptional events. Nat Rev Genet.

[3] Coppin L, Leclerc J, Vincent A, Porchet N, Pigny P (2018). Messenger RNA Life-Cycle in Cancer Cells: Emerging Role of Conventional and Non-Conventional RNA-Binding Proteins?. IJMS.

[4] Zhang B, Babu KR, Lim CY (2020). A comprehensive expression landscape of RNA-binding proteins (RBPs) across 16 human cancer types. RNA Biology.

[5] Kechavarzi B, Janga S (2014). Dissecting the expression landscape of RNA-binding proteins in human cancers. Genome Biol.

[6] Pereira B, Billaud M, Almeida R (2017). RNA-Binding Proteins in Cancer: Old Players and New Actors. Trends in Cancer.

[7] Lukong KE, Chang K, Khandjian EW, Richard S (2008). RNA-binding proteins in human genetic disease. Trends in Genetics.

[8] Kang D, Lee Y, Lee J-S (2020). RNA-Binding Proteins in Cancer: Functional and Therapeutic Perspectives. Cancers.

[9] Qin H, Ni H, Liu Y (2020). RNA-binding proteins in tumor progression. J Hematol Oncol.

[10] Meng L, He X, Zhang X (2020). Predicting the Clinical Outcome of Lung Adenocarcinoma Using a Novel Gene Pair Signature Related to RNA-Binding Protein. Gagat M, ed. BioMed Research International.

[11] Wu Y, Liu Z, Wei X (2021). Identification of the Functions and Prognostic Values of RNA Binding Proteins in Bladder Cancer. Front Genet.

[12] Li W, Li X, Gao L-N, You C-G (2020). Integrated Analysis of the Functions and Prognostic Values of RNA Binding Proteins in Lung Squamous Cell Carcinoma. Front Genet.

[13] Wang ET, Cody NAL, Jog S (2012). Transcriptome-wide Regulation of Pre-mRNA Splicing and mRNA Localization by Muscleblind Proteins. Cell.

[14] Pascual M, Vicente M, Monferrer L, Artero R (2006). The Muscleblind family of proteins: an emerging class of regulators of developmentally programmed alternative splicing. Differentiation.

[15] Wang ET, Ward AJ, Cherone JM (2015). Antagonistic regulation of mRNA expression and splicing by CELF and MBNL proteins. Genome Res.

[16] Rau F, Freyermuth F, Fugier C (2011). Misregulation of miR-1 processing is associated with heart defects in myotonic dystrophy. Nat Struct Mol Biol.

[17] Sznajder ŁJ, Michalak M, Taylor K (2016). Mechanistic determinants of MBNL activity. Nucleic Acids Res.

[18] Kanadia RN, Urbinati CR, Crusselle VJ (2003). Developmental expression of mouse muscleblind genes Mbnl1, Mbnl2 and Mbnl3. Gene Expression Patterns.

[19] Fardaei M (2002). Three proteins, MBNL, MBLL and MBXL, co-localize in vivo with nuclear foci of expanded-repeat transcripts in DM1 and DM2 cells. Human Molecular Genetics.

[20] Hao M, Akrami K, Wei K (2008). Muscleblind-like 2 (Mbnl2) -deficient mice as a model for myotonic dystrophy. Dev Dyn.

[21] Lee K, Li M, Manchanda M (2013). Compound loss of muscleblind-like function in myotonic dystrophy. EMBO Mol Med.

[22] Thomas JD, Sznajder ŁJ, Bardhi O (2017). Disrupted prenatal RNA processing and myogenesis in congenital myotonic dystrophy. Genes Dev.

[23] Batra R, Charizanis K, Manchanda M (2014). Loss of MBNL Leads to Disruption of Developmentally Regulated Alternative Polyadenylation in RNA-Mediated Disease. Molecular Cell.

[24] Lee K-Y, Chang H-C, Seah C, Lee L-J (2019). Deprivation of Muscleblind-Like Proteins Causes Deficits in Cortical Neuron Distribution and Morphological Changes in Dendritic Spines and Postsynaptic Densities. Front Neuroanat.

[25] Itskovich SS, Gurunathan A, Clark J (2020). MBNL1 regulates essential alternative RNA splicing patterns in MLL-rearranged leukemia. Nat Commun.

[26] Voss DM, Sloan A, Spina R, Ames HM, Bar EE (2020). The Alternative Splicing Factor, MBNL1, Inhibits Glioblastoma Tumor Initiation and Progression by Reducing Hypoxia-Induced Stemness. Cancer Res.

[27] Ray D, Yun YC, Idris M (2020). A tumor-associated splice-isoform of MAP2K7 drives dedifferentiation in MBNL1-low cancers via JNK activation. Proc Natl Acad Sci USA.

[28] Fish L, Pencheva N, Goodarzi H, Tran H, Yoshida M, Tavazoie SF (2016). Muscleblind-like 1 suppresses breast cancer metastatic colonization and stabilizes metastasis suppressor transcripts. Genes Dev.

[29] Tang L, Zhao P, Kong D (2019). Muscleblind-like 1 destabilizes Snail mRNA and suppresses the metastasis of colorectal cancer cells via the Snail/E-cadherin axis. Int J Oncol.

[30] Perron G, Jandaghi P, Solanki S (2018). A General Framework for Interrogation of mRNA Stability Programs Identifies RNA-Binding Proteins that Govern Cancer Transcriptomes. Cell Reports.

[31] Fischer S, Liddo Di A, Taylor K (2020). Muscleblind-like 2 controls the hypoxia response of cancer cells. RNA.

[32] Lee Y-H, Jhuang Y-L, Chen Y-L, Jeng Y-M, Yuan R-H (2016). Paradoxical overexpression of MBNL2 in hepatocellular carcinoma inhibits tumor growth and invasion. Oncotarget.

[33] Zhang J, Zheng Z, Wu M (2019). The natural compound neobractatin inhibits tumor metastasis by upregulating the RNA-binding-protein MBNL2. Cell Death Dis.

[34] Cai J, Wang N, Lin G (2021). MBNL2 Regulates DNA Damage Response via Stabilizing p21. IJMS.

[35] Esquela-Kerscher A, Slack FJ (2006). Oncomirs - microRNAs with a role in cancer. Nat Rev Cancer.

[36] Mendell JT (2005). MicroRNAs: Critical Regulators of Development, Cellular Physiology and Malignancy. Cell Cycle.

[37] Hirata H, Ueno K, Shahryari V (2013). MicroRNA-182-5p Promotes Cell Invasion and Proliferation by Down Regulating FOXF2, RECK and MTSS1 Genes in Human Prostate Cancer. Pal S, ed. PLoS ONE.

[38] Xue J, Zhou A, Wu Y (2016). miR-182-5p Induced by STAT3 Activation Promotes Glioma Tumorigenesis. Cancer Res.

[39] Lu J-T, Tan C-C, Wu X-R (2020). FOXF2 deficiency accelerates the visceral metastasis of basal-like breast cancer by unrestrictedly increasing TGF-β and miR-182-5p. Cell Death Differ.

[40] Cao M-Q, You A-B, Zhu X-D (2018). miR-182-5p promotes hepatocellular carcinoma progression by repressing FOXO3a. J Hematol Oncol.

[41] Li Y, Zhang H, Li Y (2018). MiR-182 inhibits the epithelial to mesenchymal transition and metastasis of lung cancer cells by targeting the Met gene. Mol Carcinog.

[42] Kong W-Q, Bai R, Liu T (2012). MicroRNA-182 targets cAMP-responsive element-binding protein 1 and suppresses cell growth in human gastric adenocarcinoma: MiR-182 targets CREB1 in gastric cancer. FEBS Journal.

[43] Duan L, Yan Y, Wang G, Xing Y, Sun J, Wang L (2020). ΜiR-182-5p functions as a tumor suppressor to sensitize human ovarian cancer cells to cisplatin through direct targeting the cyclin dependent kinase 6 (CDK6). J BUON.

[44] Yan S, Wang H, Chen X (2020). MiR-182-5p inhibits colon cancer tumorigenesis, angiogenesis, and lymphangiogenesis by directly downregulating VEGF-C. Cancer Letters.

[45] Newell-Price J, Clark AJL, King P (2000). DNA Methylation and Silencing of Gene Expression. Trends in Endocrinology & Metabolism.

[46] Pollex T, Heard E (2012). Recent advances in X-chromosome inactivation research. Current Opinion in Cell Biology.

[47] Li E, Beard C, Jaenisch R (1993). Role for DNA methylation in genomic imprinting. Nature.

[48] Laurent L, Wong E, Li G (2010). Dynamic changes in the human methylome during differentiation. Genome Research.

[49] Jones PA, Liang G (2009). Rethinking how DNA methylation patterns are maintained. Nat Rev Genet.

