# Supplementary material for: RNA-binding Protein MBNL2 regulates Cancer Cell Metastasis through MiR-182-MBNL2-AKT Pathway
Source: J Cancer. 2021 Sep 21;12(22):6715–26. doi: 10.7150/jca.62816 (PMC8518006; doi:10.7150/jca.62816)

Supplementary Figure legends:

Figure S1. Metascape analysis of MBNL2 correlated genes in cancer. Metascape depicts top enriched clusters and their enrichment patterns across multiple gene lists (MBNL2 correlated genes in BRCA, LUAD and LIHC, data from GEPIA) as a clustered heatmap.

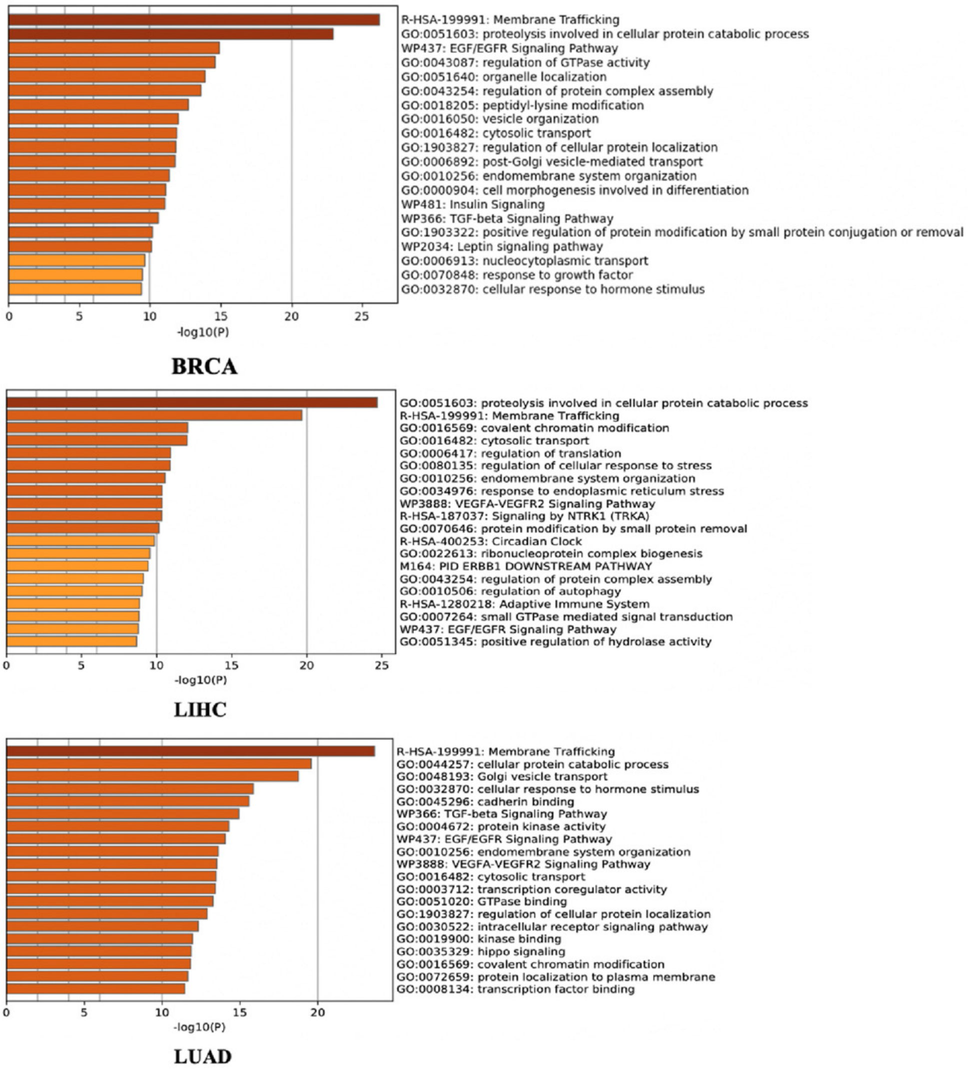

Figure S2. Depletion of MBNL2 promotes cancer cell migration and invasion in HepG2 cells. (A) qRT-PCR and immunoblotting analysis of MBNL2 expression in shCON and shMBNL2 cells. *p<0.05. (B) Migration and invasion assay showing that depletion of MBNL2 increased the number of migrated and invaded cells. ****p<0.0001.

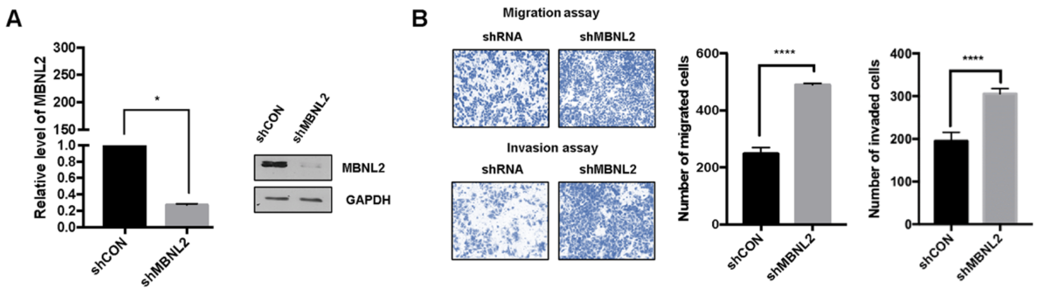

Supplement: Supplementary file 1 — Supplementary figures. [file jcav12p6715s1.pdf]
